# Cell Heterogeneity Analysis in Single-Cell RNA-seq Data Using Mixture Exponential Graph and Markov Random Field Model

**DOI:** 10.1155/2021/9919080

**Published:** 2021-05-22

**Authors:** Yishu Wang, Xuehan Tian, Dongmei Ai

**Affiliations:** ^1^School of Mathematics and Physics, University of Science & Technology Beijing, China; ^2^School of Mathematics and Statistics, Qingdao University, China; ^3^Basic Experimental Center of Natural Science, University of Science and Technology Beijing, China

## Abstract

Advanced single-cell profiling technologies promote exploration of cell heterogeneity, and clustering of single-cell RNA (scRNA-seq) data enables discovery of coexpression genes and network relationships between genes. In particular, single-cell profiling of circulating tumor cells (CTCs) can provide unique insights into tumor heterogeneity (including in triple-negative breast cancer (TNBC)), while scRNA-seq leads to better understanding of subclonal architecture and biological function. Despite numerous reports suggesting a direct correlation between circulating tumor cells (CTCs) and poor clinical outcomes, few studies have provided a thorough heterogeneity characterization of CTCs. In addition, TNBC is a disease with not only intertumor but also intratumor heterogeneity and represents various biological distinct subgroups that may have relationships with immune functions that are not clearly established yet. In this article, we introduce a new scheme for detecting genotypic characterization of single-cell heterogeneities and apply it to CTC and TNBC single-cell RNA-seq data. First, we use an existing mixture exponential family graph model to partition the cell-cell network; then, with the Markov random field model, we obtain more flexible network rewiring. Finally, we find the cell heterogeneity and network relationships according to different high coexpression gene modules in different cell subsets. Our results demonstrate that this scheme provides a reasonable and effective way to model different cell clusters and different biological enrichment gene clusters. Thus, using different internal coexpression genes of different cell clusters, we can infer the differences in tumor composition and diversity.

## 1. Introduction

Cells in the same tissue are commonly viewed as identical functional units. The analysis of traditional detection methods is always based on the overall average reaction of cells [1]. However, it has been suggested that the system-level function of a tissue is produced by heterogeneous cells between which there is a slight difference. Particularly in cancer cells, there is phenotypic and functional heterogeneity even in the same tumor [2]. The functional heterogeneity of cancer cells within tumors merits careful consideration in the conceptual history of metastasis, which involves weak and varying genetic expression between cells, or different functional cell subpopulations [3]. Traditional sequencing is always based on the average reaction of cells, so it is difficult to detect the difference. Sequencing studies on bulk tumor tissue can only identify the average gene expression. One basic aspect of cancer cell heterogeneity in the same tumor is the different levels of gene expression. By sequencing the transcriptomes of single cells in depth, low-abundance mutations can be detected that facilitate cancer classification and identification of cell heterogeneity. Recent advances have enabled the analysis of DNA and RNA within a single cell. Single-cell RNA-Seq technology is feasible and reproducible for gene expression-based classification of cell subpopulations [4–7]. Zhang et al. have demonstrated that scRNA-seq allows researchers to study the heterogeneity of gene expression in individual cells [8]. Here, we leverage the power of single-cell RNA-seq to identify individual cells with specific genetic alterations or genomic expression profiles that could be responsible for treatment resistance.

In metastatic pancreatic cancer research, the significance of CTCs in selecting appropriate therapies, monitoring therapeutic response, and innovating new treatments has been widely recognized. The heterogeneity and rarity of CTCs warrant the use of single-cell technologies to provide us with a more comprehensive understanding of these cells. Moreover, triple-negative breast cancer (TNBC) is a special type of breast cancer which represents various clinical and biological subgroups that have not yet been clearly defined [9]. Intertumor heterogeneity denotes patients who suffer from the same type of cancer but have greatly different gene expression patterns, which may be related to the tumor immune system. Single-cell RNA sequencing technology has been used to explain tumor microenvironment heterogeneity by identifying distinct cell subsets that may be associated with immunosurveillance and are potential immunotherapy targets.

However, large-scale data is a significant obstacle to obtaining the highest-resolution analysis of intracell genetic heterogeneity, due to the data complexity of scRNA-seq datasets. Recent research on heterogeneity analysis has focused almost completely on using clustering algorithms (such as PCA, SVM, and hierarchical clustering) to find modularity in gene expression [10, 11]. Wang et al. [12] have reviewed the methods and tools that dedicate to the different task and usages. They also provided a guide to utilize scRNA-seq technology [8]. Although these methods have achieved impressive results, as gene expression data and module complexity increase, traditional clustering algorithms have difficulty discovering the different expression modules. The corresponding computational problem has fewer objects (cells) than the number of variables (genes). Usually only a few out of 1000 genes are significantly differentially expressed in distinct cell types, which reduces the effectiveness of traditional models. Because when clustering on the whole transcriptome, many genes would be regarded as irrelevant attributes and may even impede the identification of cell types. It has been claimed that for a broad range of data distributions, the conventional similarities (such as Euclidean norm or Cosine measure) become less reliable as the dimensionality increases [13]. The reason is that all data become sparse in high dimensional space, and therefore, the similarities between objects measured by these metrics are generally low. This inspired us to propose a more favorable network clustering algorithm to uncover additional unknown genetic changes and cellular states, which would normally be regarded as irrelevant attributes.

Graphical models bring together graph theory and probability theory in a powerful formalism for multivariate statistical modeling. The key idea is factorization: the collection of probability distributions that factorize according to the structure of an underlying graph. Inspired by this ideology, if single cells construct a network, one can use a graphical model to divide this graph. One of the most deliberate graphical models is the exponential random graph model (ERGM) [14], which takes into consideration the probability distribution of the existing network ensuing from the exponential family to model the edge distribution of the existing graph. But, the ERGM model on its own cannot represent the clustering feature of the network. In this study, we adopted a mixture ERGM model proposed by Wang et al. [1], which extends the latent space model to take account of the clustering feature and identify single-cell RNA-seq data for different cell subtypes.

By representing data as a relationship graph in which nodes correspond to data points and edges (valued as 0 or 1) represent the relationships between data points, the graph can be partitioned into homogeneous and well-separated subgraphs to achieve the clustering task. Regarding the single-cell RNA-seq data, we first calculated the Pearson correlation coefficients (PCCs) among all the cells pairwise, based on their mRNA expressing profile. More specifically, we used Fisher's method to test the significance of the difference between PCCs (see Materials and Methods). We thus obtained a cell-cell network with valued edges (0 or 1).

However, in the original MixtureERGM model, subnetworks were based on the hypothesis that edges between nodes from different two subnetworks arise randomly. We found that this assumption was not accurate enough for the gene coexpression network. Because gene subnetworks denote different functional assemblies or gene pathways, there are usually latent relationships between different subnetworks, such as hub nodes or functional genes. Based on this, we improved the original MixtureERGM model by introducing forms of dependence between subnetworks with a Markov structure. That is, using the MixtureERGM model, when given the network structure and node classifications grouped by MixtureERGM, the posterior probability of the intercluster network configured by the Markov random field model can be inferred through a Bayesian framework. Meanwhile, there are two advantages to the Markov random field model. First, the model can incorporate network structures, which account for long-distance dependencies in associate states. Second, the computational framework with the Monte Carlo Markov chain is well established. In addition, we proposed an online EM algorithm for our MixtureERGM model which can solve the computation challenge for large networks. Actually, online parameter estimation using mixture models has already been studied by [15, 16].

We downloaded the single-cell RNA-seq data from Ting et al. [17] and Wang et al. [18]. These two scRNA-seq datasets are for pancreatic CTCs and triple-negative breast cancer (TNBC), respectively, and both focus on defining subsets of tumors with different molecular characterizations and finding the highly differentially expressed genes. Studies of bulk sequencing populations cannot resolve the degree of heterogeneity across these poorly understood cell populations. In the original studies connected with these datasets, cell types corresponding to each cell cluster were inferred based on prior knowledge about type-specific marker genes and the clustering results of gene expressions. In the present study, our scheme based on MixtureERGM and the MRF model provided powerful technical support for mining biological information in gene expression data and revealing the heterogeneity of gene expression between different tumor cells. Furthermore, we found various expressing genes and enriched GO functional patterns which helped us to determine the functions of cell subgroups.

Because our research was focused on the network clustering for scRNA-seq analysis, in order to demonstrate the effectiveness of our methodology for identifying cell types, we compared it with another network clustering algorithm proposed by Salter et al. [13], on one synthetic dataset and two real scRNA-seq datasets. To avoid the simulation setup favoring our own model, we generated synthetic dataset from [13].

## 2. Materials and Methods

### 2.1. Cell-Cell Network

To obtain cell clustering information and determine different gene coexpression patterns in different cell subgroups, we first transformed the single-cell gene expression data into a cell-cell network. We excluded the edges between cells if the Pearson correlation coefficient between two cell data arrays in the gene expression matrix was cor_Pearson correlation_ < 0.27, which corresponds to the 0.95 quantile of the Student *t*-distribution. In Eq. (1), *n*_*m*_ is the number of gene samples and *r* is the Pearson correlation coefficient (PCC). Otherwise, one edge was selected to represent a relationship between the two cells.

(1) $$\begin{array}{c} T=r\sqrt{\frac{n_{m}-2}{1-r^{2}}}\sim t_{n_{m}-2}. \end{array}$$

### 2.2. MixtureERGM Model

In the network, each node is a cell, with an adjacent matrix *Y*, where *y*_*i*,*j*_ denotes the value of the relationship between nodes *i* and *j*. *y*_*i*,*j*_ = 1 denotes an edge between nodes *i* and *j*. In the ERGM model, the probability of one observed network *Y* is proportional to the exponent of the sum of the network statistics multiplied by some parameters:

(2) $$\begin{array}{c} P\left(\left.Y\right|\theta \right)=\exp\left(\theta^{T}\left(S\left(Y\right)\right)-\gamma \left(\theta \right)\right), \end{array}$$

where *θ* is the parameters of the model, *S* (*Y*) are network summary statistics chosen by the analyst, and *γ*(*θ*) is a normalization constant (also called a partition function in statistical physics).

We introduce unobserved indicator variables *Z*_*i*_ as the class vector for every node classification, following a multinomial distribution:

(3) $$\begin{array}{c} Z_{i}=\left(Z_{i1},Z_{i2},\cdots ,Z_{iG}\right)\sim \text{M}\left(1,\alpha_{1},..,\alpha_{g}\right), \end{array}$$

where the latent variable *Z*_*ig*_=1 if node *i* belongs to class *g* and zero otherwise.

Then, we assume that the network of each subgroup of cells with the attached edges fits a finite ERGM model and has a specific parameter vector *θ*_*g*_. The probability of network *Y*_*g*_ given the classification of nodes is as follows:

(4) $$\begin{array}{c} P_{\emptyset }\left(Y\;|\;Z\right)=\underset{q,l,i,j}{\prod }{\left[\exp\left\{\theta_{l,q}^{T}{\left(S\left(Y\right)\right)}_{ij}-\gamma \left(\theta_{l,q}\right)\right\}\right]}^{Z_{iq}Z_{jl}}, \end{array}$$

where *θ*_*l*,*q*_ is the parameter of ERGM， and (*S*(*Y*))_*ij*_ is the sum of network statistics calculated by the analyst, such as edges, geometrically weighted in-degree distribution, geometrically weighted out-degree distribution, mixed 2-stars, and triangles. According to Cho et al. [9], the latent variable  *Z*_*iq*_ = 1 if node *i* belongs to class *g* and zero otherwise. Since we focused on finding the clustering results of mixture ERGMs, we tried to select network statistics, such as the differences of network attributes of nodes, with the attached edges inside or outside one cluster. In order to infer the properties of subnetworks, we selected the terms of ERGM in one cluster, including the following: edges, geometrically weighted in-degree distribution, geometrically weighted out-degree distribution, and mixed 2-stars. So, the joint probability of network *Y* under given conditions *Z* is as follows:

(5) $$\begin{array}{c} P_{\emptyset }\left(Y,Z\right)=P_{\emptyset }\left(Z\right)P_{\emptyset }\left(Y\;|\;Z\right)=\underset{i,q}{\prod }\alpha_{i}^{Z_{iq}}\underset{q,l,i,j}{\prod }{\left[\exp\left\{\theta_{l,q}^{T}{\left(S\left(Y\right)\right)}_{iq}-\gamma \left(\theta_{l,q}\right)\right\}\right]}^{Z_{il}Z_{jl}}. \end{array}$$

The classifications of nodes and parameter estimation can be inferred with an iterated online EM algorithm [1].

### 2.3. Markov Random Field Modeling Approach

After exploiting the network features with the MixtureERGM model, we obtained the node classifications and network joint optimal probability distribution simultaneously. Nevertheless, in order to take the intercluster relationships into consideration by prioritizing hub nodes, we introduced a new indicator value *hub value*: *hv*_*i*_ = *n*_*i*_/degree_*i*_ for each node *i* , where *n*_*i*_ is the number of subgroups attached with node *i*, and degree_*i*_ is the degree of node *i* in the network. Then, we normalized the hub value to a range between 0 and 1. We utilized a Gaussian Markov random field model to formulate the intercluster network probability. Under the null hypothesis of no hub node, each hub value has a uniform (0,1) distribution. In this paper, we consider *ω* = (*ω*_1_, *ω*_2_, ⋯., *ω*_*n*_), where *ω*_*i*_ = *Φ*^−1^(1 − *hv*_*i*_/2) and *Φ*(.) are the CDF (Cumulative Distribution Function) of *N*(0, 1). Define the state of node *i* by *T*_*i*_ = 1  (*H*_*i*0_ is false) if node *i* is the hub node; that is, *T*_*i*_ = 1 or *T*_*i*_ = 0 corresponds to whether *H*_*i*1_ or *H*_*i*0_ holds. Then, the null distribution of *ω*_*i*_  will be exactly the standard normal (Eq. (6)). Under the alternative hypothesis, i.e., the node state is not a hub node, *T*_*i*_ = 0, we follow *Chen* et al. [19] by assuming the distribution (Eq. (7)).

(6) $$\begin{array}{c} P\left(\omega_{i}\;|\;T_{i}=0\right)∼N\left(0,1\right), \end{array}$$

(7) $$\begin{array}{c} P\left(\omega_{i}\;|\;T_{i}=1\right)∼N\left(\mu_{i},\sigma_{i}^{2}\right), \end{array}$$

and $\mu_{i}|\sigma_{i}^{2}∼N\left(\bar{u},\sigma_{i}^{2}/a\right)$,*σ*_*i*_^2^ ~ InverseGamma(*υ*/2, *υd*/2).

The distribution of network configuration is defined as follows:

(8) $$\begin{array}{c} P\left(T_{1},\cdots ,T_{n}\;|\;\theta_{0}\right)=\frac{1}{Z\left(\theta_{0}\right)}\exp\left(h\underset{i}{\sum }I_{1}\left(T_{i}\right)+\tau_{0}\underset{<i,j>\in \varepsilon }{\sum }\left(d_{i}+d_{j}\right)I_{0}\left(T_{i}\right)I_{0}\left(T_{j}\right)+\tau_{1}\underset{<i,j>\in \varepsilon }{\sum }\left(d_{i}+d_{j}\right)I_{1}\left(T_{i}\right)I_{1}\left(T_{j}\right)\right), \end{array}$$

where *θ*_0_ = (*hτ*_0_, *τ*_1_) are the prior parameters or hyperparameters, *I*_0_(.) and *I*_1_(.) are the indicator functions, *d*_*i*_ = degree_*i*_^1/2^, and *Z*(*θ*_0_) is a normalizing function that is summed over all 2^*n*^ possible configurations.

Given the network structure and the node classification grouped by the MixtureERGM algorithm, the posterior probability of the intercluster network configuration can be inferred with a Bayesian framework:

(9) $$\begin{array}{c} P\left(T\;|\;\omega ,\theta_{0}\right)\propto P\left(\omega \;|\;T\right)P\left(T\;|\;\theta_{0}\right). \end{array}$$

The inference of labels and parameters are according to the posterior distribution of *T*:

(10) $$\begin{array}{c} \hat{\text{T}}=\underset{T}{\text{argmax}}P\left(\omega \;|\;T\right)P\left(T\;|\;\theta_{0}\right). \end{array}$$

A Gibbs sampler as outlined above can be applied to stochastically search for the solution to the above optimization problem [20].

### 2.4. Gene Set Enrichment Analysis

Suppose that there are *M* genes in the background set, and *m* of those genes is prioritized. The number of overlap genes between the background set and the prioritized set with a functional gene set is *M*_*p*_ and *m*_*p*_, respectively. In the hypergeometric test, the enrichment *P* value was calculated as follows:

(11) $$\begin{array}{c} P=\frac{C_{M_{p}}^{m_{p}}C_{M-M_{p}}^{m-m_{p}}}{C_{\text{M}}^{m}}. \end{array}$$

### 2.5. Choosing the Number of Clusters

The integrated classification likelihood (ICL) is used to choose the optimal number of classes, as explained in [21]. This strategy was carried out by running the MixtureERGM algorithm from 2 to Q classes and selecting the solution which maximized the ICL criterion (Q can be decided by the researchers). The ICL criterion can be defined as follows:

(12) $$\begin{array}{c} \text{ICL}\left(G\right)=-2L_{C}\left(X,Z,\emptyset \right)+\left(GM+G-1\right)\log\left(n\right), \end{array}$$

where *L*_*C*_ is the value of the classification log-likelihood, *G* is the number of groups, and *M* is the number of summary statistics in the model.

## 3. Results

### 3.1. Simulation Results

We simulated one undirected network from three ERGM types using sufficient network statistics: the number of edges and m2stars, the geometrically weighted edgewise shared partner distribution, and the geometrically weighted degree distribution. These three ERGMs formed three separate clusters, the parameters of which were generated:

We then applied our algorithm and the role analysis algorithm [13] to fit a mixture of ERGMs. When we ran this experiment 50 times, the averaged parameters estimated by these two algorithms were as follows:

(13) $$\begin{array}{c} {\bar{\alpha }}_{\text{role}}=\left[\begin{array}{c} 0.8\\ 0.15\\ 0.05 \end{array}\right]{\bar{\theta }}_{\text{role}}=\left[\begin{array}{c} 1.55\;\\ -18.1\\ -35.2 \end{array}\begin{array}{c} 0.9\\ 1\\ -7.6 \end{array}\begin{array}{c} 0.08\;\\ 5.8\;\\ 4.5 \end{array}\begin{array}{c} 0\\ 10\\ 4.8 \end{array}\right],\\ {\bar{\alpha }}_{\text{MixtureERGM}}=\left[\begin{array}{c} \frac{3}{11}\\ \frac{4}{11}\\ \frac{4}{11} \end{array}\right]{\bar{\theta }}_{\text{MixtureERGM}}=\left[\begin{array}{c} 1.2\;\\ -1.2\\ 18 \end{array}\begin{array}{c} 0.5\\ -1\\ -7.6 \end{array}\begin{array}{c} -1.3\;\\ -2.3\;\\ 5 \end{array}\begin{array}{c} -1\\ 10\\ 7 \end{array}\right]. \end{array}$$

**Figure 1:**
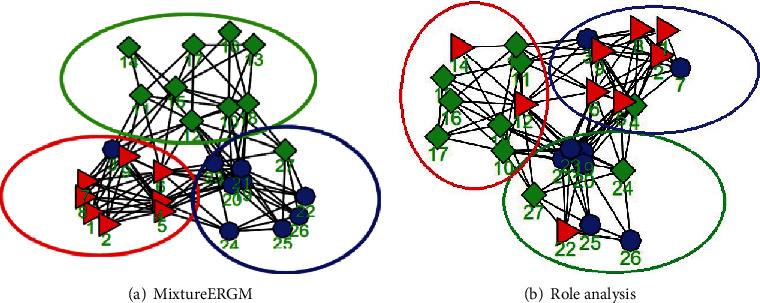
Clustering results with three groups of synthetic dataset by (a) MixtureERGM and (b) role analysis, where the original groups are denoted by the different color circles and grouping results by algorithm are denoted by the different color nodes.

This showed that our method estimated much more accurately than role analysis. The method was also better at clustering the ERGM networks and estimating ERGM parameters in the synthetic dataset. On the other hand, Figure 1 shows the clustering of the synthetic dataset by the two models. It is evident that the clustering results of MixtureERGM almost agreed with the ground truth, while the role analysis nearly clustered into one group.

### 3.2. CTC scRNA-seq Datasets

We applied the MixtureERGM model and MRF approach to the two single-cell RNA-seq datasets. The first one was from mouse pancreatic circulating tumor cells, from Ting et al. [17], containing 149 cells and 19,681 genes; the second was from triple-negative breast cancer, from Wang et al. [18], containing 1534 cells and 21785 genes.

#### 3.2.1. Pancreatic CTCs

Circulating tumor cells (CTCs) are shed from primary tumors into the bloodstream, mediating the hematogenous spread of cancer to distant organs. Analyzing the CTC RNA-seq enabled us to define and classify the subsets of CTCs with different highly expressed marker genes.

**Figure 2:**
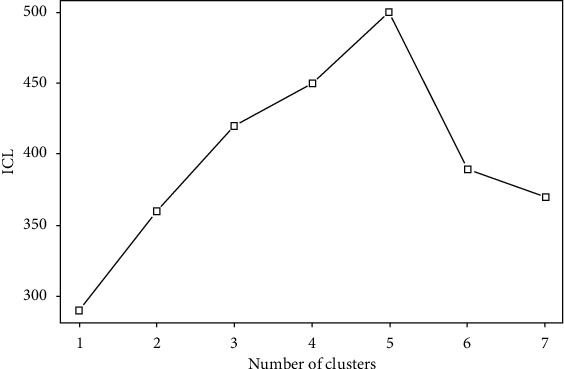
The plot of ICL of MixtureERGM algorithm against number of clusters for CTC dataset.

To construct a more meticulous interrelationship network of pancreatic circulating tumor single cells and cell heterogeneity from the network angle, we first constructed a cell-cell network according to this single-cell RNA expressing profile and applied the MixtureERGM model to it, resulting in five cell clusters. ICL of the MixtureERGM algorithm led to selection of 3 groups (Figure 2). Meanwhile, we compared the results from our methodology and from role analysis in detecting the number of significant enrichment functional GO items (*P* value <0.05) (see Table 1(a)).

**Figure 3:**
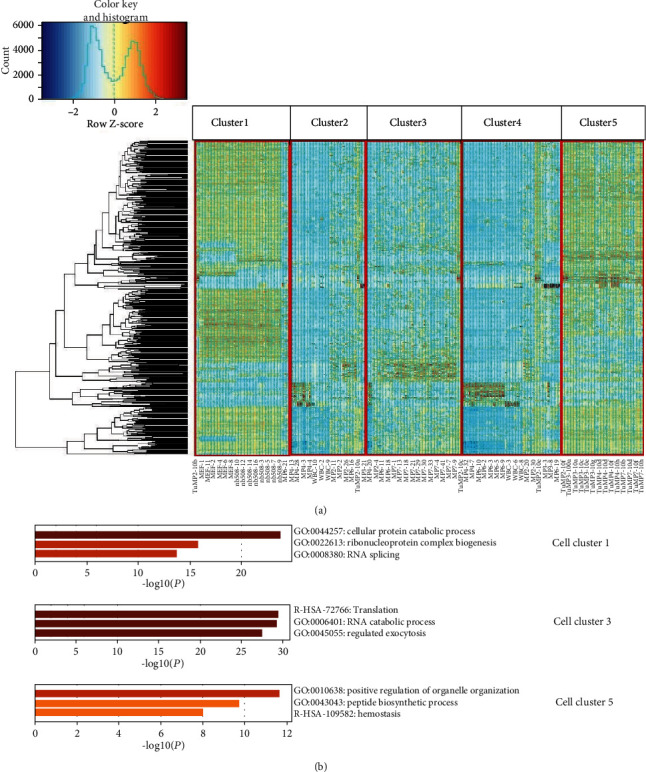
(a) Gene expression profiles of circulating tumor cells were clustered using MixtureERGM algorithm with 5 underlying clusters. Each column represents one cell. (b) GO enrichment results of high coexpression genes in these three cell clusters generated by MixtureERGM algorithm. *P* values are denoted by the color bars.

Figure 3(a) gives the clustering results of MixtureERGM, in which there were five cell clusters, three of which had significant GO functional enrichment. These were cell clusters 1, 3, and 5 in our clustering results, where cluster 1 was consistent with pancreatic dual adenocarcinoma (PDAC) cell lines (*P* value of GO enrichment was 3.12 × 10 − 15), cluster 3 was consistent with the classical CTCs (*P* value of GO enrichment was 5.36 × 10 − 16), and cluster 5 was consistent with the primary tumor cells with Ting et al. (*P* value of GO enrichment was 7.42 × 10 − 17).  Figure 1(b) gives the high coexpression gene GO enrichment items.

**Figure 4:**
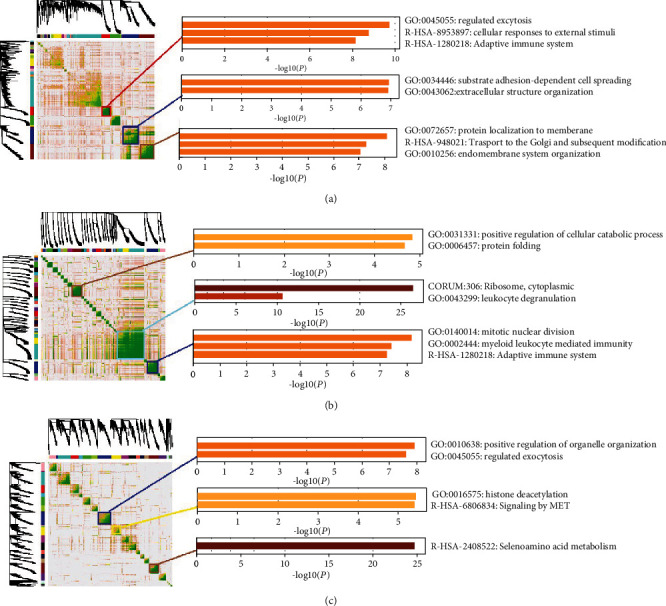
Coexpression gene modules in cell cluster 1 (A), cell cluster 3 (B), and cell cluster 5 (C). Yellow bars indicate the negative log of *P* values.

In order to explore the detailed coexpression gene module in different cell types, we adopted the WGCNA approach for these three clusters.  Figure 4 gives the heat map results of the coexpression gene patterns and the biologically significant results in these three cell populations, where yellow bars indicate the negative log of *P* values in formula (11). In this figure, we can see that the high-expression genes in cell cluster 1 mainly participated in functions that regulated exocytosis, cellular responses to external stimuli, extracellular structure organization, and transport to the Golgi, in addition to subsequent modification. Meanwhile, the high-expression genes in cell cluster 3 mainly participated in positive regulation of the cellular catabolic process and protein folding, ribosome leukocyte- and myeloid leukocyte-mediated immunity, and the adaptive immune system, which is consistent with its cell category. Furthermore, the high-expression genes in cell cluster 5 mainly participated in positive regulation of organelle organization, histone deacetylation, and selenoamino acid metabolism, which are all important functions in primary tumor development.

**Figure 5:**
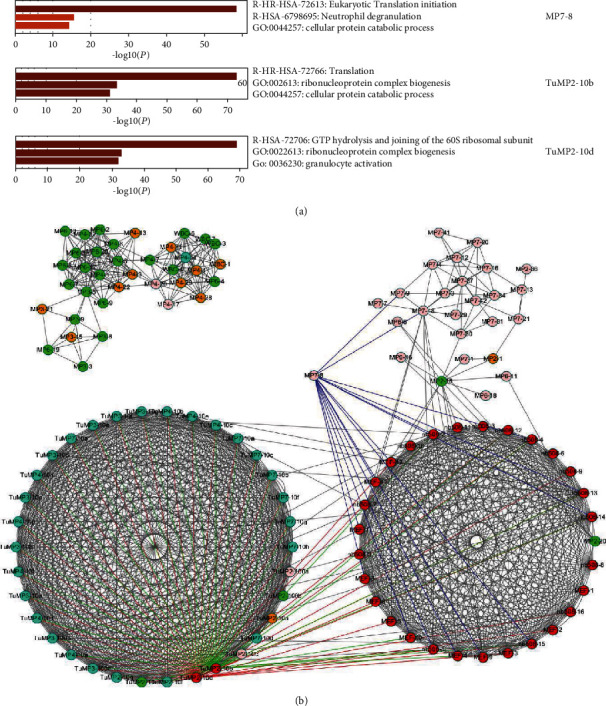
Hub nodes were generated by MRF algorithm, green nodes denote cell cluster1, red nodes denote cell cluster 3, and light blue nodes denote cell cluster 5. (a) Negative log of *P* values when calculating the significance of GO enrichment functions of high-expression genes in these hub cells are indicated by yellow bars.

Next, to find the hub node cells, which are important connectors among different function patterns and for internetwork rewiring information, we applied the MRF model to the classified cell-cell mixture network.  Figure 5 gives the results for hub nodes in the cell network, in which we found three important hub nodes. The hub nodes specifically expressed in immune cells were MP7-8, TuMP2-10b, and TuMP2-10d. These hub cell nodes indicated a link with translation or GTP hydrolysis, which are both important in the metabolism and evolution of tumors. The coexpression of these genes identified in some regulatory T-cell clusters may be potential immunotherapy targets. From gene expression profile, we can find that TuMP2 cell has high expressed gene KRT7, KRT8, which is functional in epithelial, and low expressed gene Cd61, which is functional in hematopoietic. Specially, gene KRT8 is also found in the triple-negative breast cancer dataset, which may be a generic cancer gene.

On the other hand, although MixtureERGM and the MRF model gave the CTC clusters and the molecular features of tumor cells, defining cell heterogeneity required additional analysis. For this, we used nonparametric differential gene expression analysis, including a rank product (RP) methodology [22] to identify relevant differentially expressed genes between two different cell clusters. CD45 is found to express differently in CTC cells and primary tumor cells.

**Figure 6:**
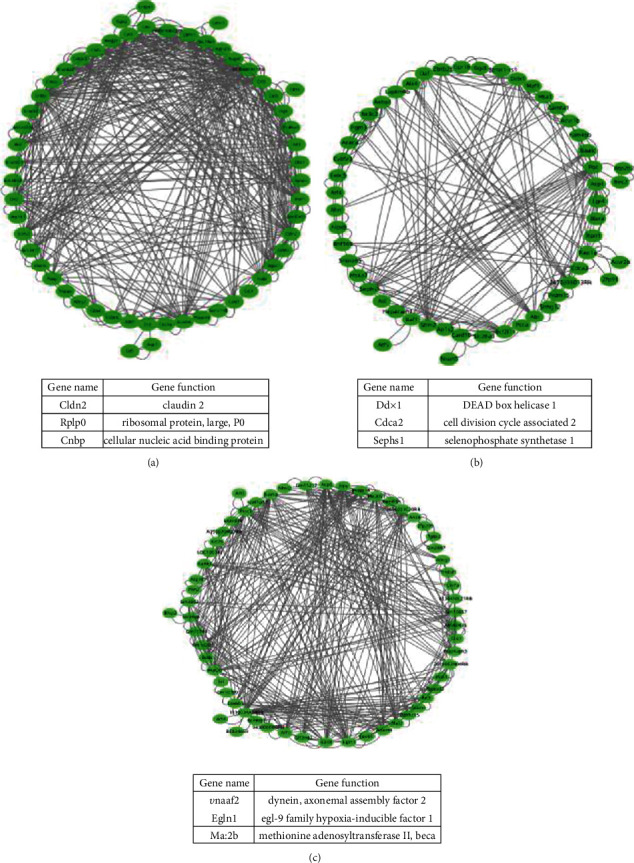
(a) Gene network of differentially expressed genes between cell clusters 3 and 5 and hub node genes. (b) Gene network of differentially expressed genes between cell clusters 1 and 3 and hub node genes. (c) Gene network of differentially expressed genes between cluster 1 and cluster 5.

The first step was to analyze the differentially expressed genes between cell clusters 3 and 5. There were 63 differentially expressed genes and 476 edges in this gene-gene network. Through MFR algorithm analysis, we found three important hub node genes. Pathway and process enrichment analysis gave the significant biology functions, expressed in (Figure 6(a)). These hub genes play an important role in protein coding. Other similar results are shown in Figures 6(b) and 6(c).

**Figure 7:**
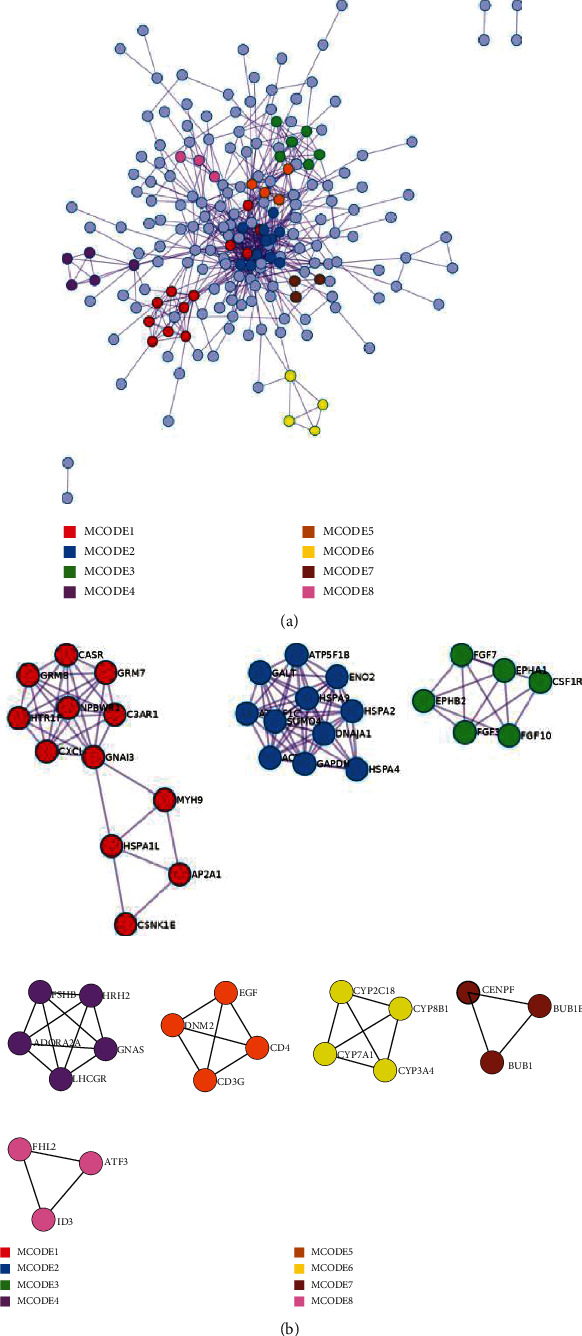
(a) Protein fully connected interaction network. Different colors denote different protein modules. (b) Enriched protein clusters in the protein-protein network translated by these differentially expressed genes.

Next, we analyzed the protein-protein interaction enrichment translated by these differentially expressed genes from CTC cell clusters and tumor cell clusters. We obtained the protein-protein network by protein-protein interaction enrichment analysis (*P* value <0.05). Then, we used a molecular complex detection (MCODE) algorithm, obtaining the function enriched protein modules shown in Figure 7. Function enrichment analysis demonstrated that these proteins were mostly involved in cancer pathways, cytokine-mediated signaling pathways, and interleukin signaling, which are all important pathways in tumor evolution. We found that the difference between CTC cell clusters and tumor cell clusters was mainly caused by these different gene functions. As indicated in that study, our method based on scRNA-seq data enabled the discovery of minor subgroups of CTC cells that were related to immunosuppression or cancer metastasis.

#### 3.2.2. Triple-negative breast cancer (TNBC)

TNBC is the most vicious subtype of breast cancer usually with bad prognosis. The identification of cell types using scRNA-seq technology promoted to identify the constitution of cell types, followed by differentially expressed genes or “marker gene” which maybe related with prognosis [12]. We downloaded the TNBC single-cell RNA-seq data, which included 1534 cells, from the GEO database (https://www.ncbi.nlm.nih.gov/geo/). Similar to the scheme for CTC data, we introduced the MixtureERGM and MRF model into this single-cell gene expression profile and obtained cell type classifications and hub nodes in the cell-cell network. We divided these 1534 cells into 15 clusters, where there were 7 GO-enriched cell types.  Table 2 gave the results of cell type functional annotations, where *P* value <0.001 (here, we adopted a more strictly hypergeometric test level than that in Table 1). We also give the comparison results with the role analysis clustering algorithm in Table 1(b).

**Figure 8:**
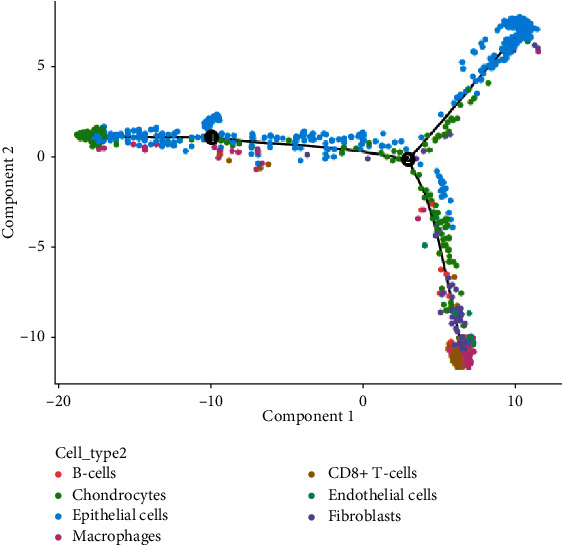
Single-cell trajectory results. Different color nodes represent different cells.

**Figure 9:**
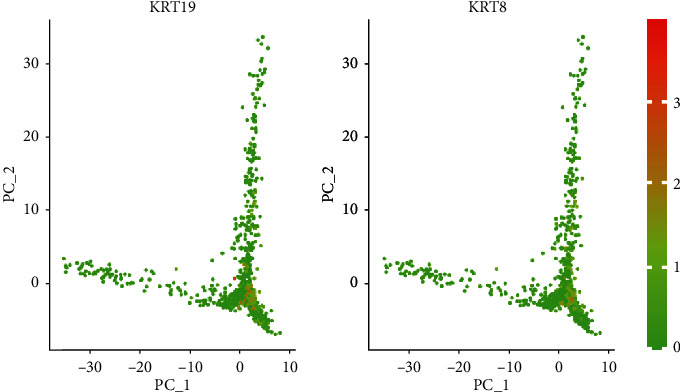
Genes “KRT19” and “KRT8” expressing pattern in different cells.

In order to introduce how cell moved through biological progress in pseudo time, we employed the Monocle algorithm proposed by Trapnell et al., for which we chose the different expressing genes among our cell clusters.  Figure 8 shows these single-cell trajectories, where CD8+ T-cell (cluster 3 in our clustering results) and macrophages (cluster 4 in our clustering results) have similar branching trajectories. This is consistent with the high coexpression gene patterns. Genes “JUNB,” “DUSP1,” “FOS,” “EGR1,” “KRT19,” “KRT8,” and “SPARC” were all marker genes in both of these clusters.  Figure 9 shows a two-dimensional projection of expressing pattern for the “KRT19” and “KRT8” genes in different cells, which illustrates the consistently high expression in the typical cell subgroups.

**Figure 10:**
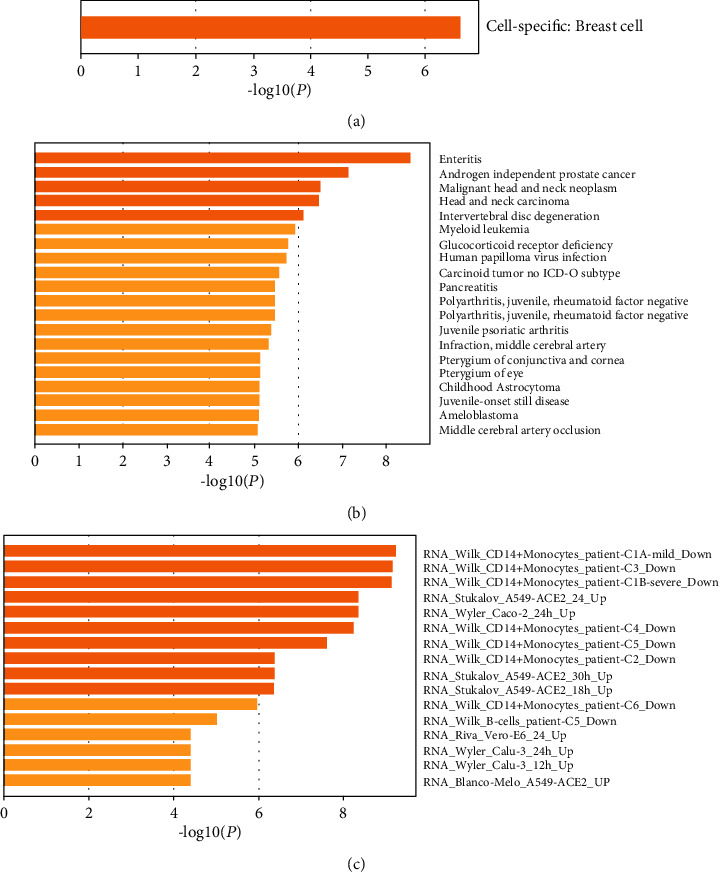
Enrichment function items of genes “JUNB,” “DUSP1,” “FOS,” “EGR1,” “KRT19,” “KRT8,” and “SPARC.” (a) Cell category in which genes high expressed, (b) comparison with the information on human disease-associated genes, and (c) comparison with COVID gene functional dataset.

The final stage was to deeply exploit these specific expressed genes. First, we performed enrichment analysis in the PaGenBase (a pattern gene database for the global and dynamic understanding of gene function) [23]. The FOS, KRT8, and KRT19 genes belong to specific breast cells (*P* value = 0.00136). Next, we adopted a comprehensive platform integrating information on human disease-associated genes—DisGeNET [24]—and found several of these genes to be closely related with other malignant tumors. For example, DUSP1, JUNB, and SPARC genes were significantly related with Endometrioid (*P* value = 0.0082); DUSP1, EGR1, FOS, and JUNB genes were significantly related with lung tumor (*P* value = 0.0074); more results are given in Figures 10(a) and 10(b).

It is worth mentioning that we also found several genes to be significantly related with COVID [25]. For example: genes DUSP1, EGR1, FOS, and JUNB are enriched in GO COVID245. These genes are functioned as RNA-Wilk-CD14+monocytes, which is related with patient-C1A-mild-down. More results could be found in Table 3. From the functional category results, we can see that most of these genes are involved in the CD14+monocyte function, which is an important role in the immune system (see Figure 10(c)).

## 4. Discussion

In this study, we introduced the MixtureERGM MRF model into single-cell RNA-seq data, demonstrating that the algorithm can perform effective clustering and simultaneously find the hub nodes in cell networks. We also compare our approach with another method of network clustering algorithm: role analysis which is focused on finding roles of nodes in networks. It extracts a network into several ego-networks, in which every node is interlinked with the others. However, this assumption would destroy the inherent correlation of one network and may amplify the conditional correlation, which is not real connections among nodes. In contrast, the MixtureERGM model considers the joint probability of the observed network proportional to the exponent of the sum of the subnetwork statistics, where *S*(*Y*)_*ij*_ are different network statistics according to the belongings of nodes *i* and *j*. For the relationships between two different subnetworks, we adopted the Hidden Markov random field model to prioritize hub nodes with network rewiring. The MixtureERGM and MRF models fit the cell-cell network with graph angle, which overcomes the high-dimension problems in single-cell RNA-seq data. RNA-seq data is generally on the scale of tens of thousands, which can greatly complicate the clustering problem.

We applied the MixtureERGM network clustering model and MRF algorithm to find the heterogeneity and hub nodes of two datasets. In the first dataset, cluster 1 is consistent with pancreatic dual adenocarcinoma (PDAC) cell lines, cluster 3 is consistent with classical CTCs, and cluster 5 is consistent with the primary tumor cells clustered by Ting et al. From the heat map (Figure 3), it is clear that genes within the same cluster have a strong correlation, while there are marked differences between genes in different clusters. Meanwhile, we used a nonparametric differential gene expression analysis including rank product (RP) methodology to identify relevant differentially expressed genes between two different cell clusters. Finally, we analyzed the protein-protein interaction enrichment translated by these differentially expressed genes from CTC cell clusters and tumor cell clusters. We found that the difference between CTC cell clusters and tumor cell clusters is mainly caused by these different gene functions. Identifying immune cell subtypes and their distribution is important to reveal immune cell infiltration patterns among different patients, which may provide an opportunity for the design of personalized treatments. With the second dataset, we obtained the trajectories for different cell types using our methodology, as well as the different expression genes across different cell clusters. Specifically, we found seven important marker genes playing important roles in the immune system, all of which were closely linked to generic cancer genes.

As the statement of Zhang et al. [8], there have been many scRNA data analysis tools, with different advantages and disadvantages. For avoiding the model-based methods heavily depending on whether the data fit the model, they present one multiple kernel combination methods, which could automatically learns similarity information from scRNA-seq data and transform the candidate solution into a new one. Different with the kernel learning method, our method was focused on clustering cells according to their network property, determined by the correlation of gene expression profiles. So we did not compare these two methods in different directions. However, some limitations of our method can be found. (i) The computation time would rapidly increase when the cell numbers are more than ten thousands. (ii) The number of clusters needs to be determined in advance, which may leading to subjective assume by researchers. In the future, we will continue improving efficiency and effectiveness of the network clustering algorithm based on characteristics of scRNA-seq data.

## Figures and Tables

**Table:** (a) Comparison results of MixtureERGM model and role analysis model in pancreatic CTC database

Method Num	MixtureERGM model	Role analysis model
Number of different expressing genes across different cell groups	236	185
Number of GO enrichment gene modules, in which numbers of genes > 20 (*P* value <0.05)	15	10

**Table:** (b) Comparison results of MixtureERGM model and role analysis model in triple-negative breast cancer (TNBC) database

Method Num	MixtureERGM model	Role analysis model
Number of different expressing genes across different cell groups	556	323
Number of GO enrichment gene modules, in which numbers of genes > 20 (*P* value <0.05)	28	21

**Table 2:** Results of cell type functional annotations found by MixtureERGM model.

Cluster ID	GO annotations
1	Epithelial cells
2	Chondrocytes
3	CD8+ T-cells
4	Macrophages
5	Fibroblasts
6	B-cells
7	Endothelial cells

**Table 3:** Genes found to be significantly related with COVID.

GO items	GO functions	*P* value	Gene names
COVID245	RNA_Wilk_CD14+monocytes_patient-C1A-mild_down	0.0001	DUSP1|EGR1|FOS|JUNB
COVID191	RNA_Stukalov_A549-ACE2_24h_up	0.00025	DUSP1|EGR1|FOS|JUNB
COVID046	RNA_Wyler_Caco-2_24h_up	0.00025	DUSP1|EGR1|FOS|JUNB
COVID253	RNA_Wilk_CD14+monocytes_patient-C4_down	0.00027	DUSP1|EGR1|FOS|JUNB
COVID189	RNA_Stukalov_A549-ACE2_18h_up	0.0017	DUSP1|EGR1|JUNB
COVID257	RNA_Wilk_CD14+monocytes_patient-C6_down	0.0025	DUSP1|FOS|JUNB
COVID346	RNA_Wilk_B-cells_patient-C5_down	0.0067	DUSP1|FOS|JUNB

## Data Availability

Datasets in this article were held in the NCBI Gene Expression Omnibus with the accession numbers GSE51372 and GSE118389.
